# 

**Published:** 1881-12

**Authors:** 


					﻿Pamphlets received.—We return thanks for the following:
“ Private Care of the Insane.1’ By Ralph L. Parsons, N. Y.
“ Sewers and Sewer Gas.” By D. H. Beckwith, Cleveland,
Ohio.
Introductory Lecture, at Pulte Med. College. By Prof. Wm.
Owens, Cincinnati.
The Address of the President of the Hom. International
Convention, held in London, July, 1881. By Prof. R. Hughes.
thstzdzezk
TO THE
Homeopathic Physician.
■V O Tj XT 2MZ ZE3 I.
Page
Abdominal Complaints.................-.......499
Abnormalities of Labor. L. B. Wells,
M. D.............—...................... 310
Aconitum Napellusin its Relation to
the Female 8exual System. C.
Carleton Smith, M. D...............„........„	569
Aeon.„100.113,115,144,154,188, 214. 264
I 268,269,313,322,335,381,382,384,390,
404,407,409,418,419,425,431,432,453,
497, 498, 500, 569, 581, 582, 583, 584, 603
Aeon. nit. gal...—........................... 212
Acroehordon Chocoe—Characteristics.
C. F. Nichols, M. D.....................444
Actsea Rae.....—..;_________...........422,431, 447
Action of Homoeopathic Globules on
Persons in Health.......................178
Address at Milwaukee, My. E. W.
' Berridge, M. D..._________....................— 46
-Esculus Hipp. ...50,144,145,188,189,190,
191,192, 265, 373, 431
^Eth_________................................ 312
Alcohol______.......—..........................580, 581
Agaricus..................338, 339,384,449, 497
Agnus C................................ 579
Aggravation from the Two Hundredth. 222
Allen, H. C., M. D. Viburnum Op. A
Fragmentary Proving....101
Aloes Socot___________144,145,152,153,192, 431
Alternation of Medicines, On the. Drs.
Martiny & Bernard....-.................. 408
Alumen ...........________......144,193,194, 422
Alumina...145, 194, 372. 375,381,383,431,
447, 497, 531
Ambra G__________.......313,381, 383, 384,450, 497
Amenorrhoea....—......................_...... 339
Ammon, carb__________.144,145,147,149,194,
292, 293, 294, 295, 296, 297, 298, 299,
300, 301, 302, 303, 304, 312, 381, 383, 578
Ammon, mur...............144,145,195,382, 578
Amyl nitrite...—_______—.....................426, 505
Anacard...............................145, 372,
381, 382, 383, 384, 394, 496, 498, 579
Anthracinum_________.................................. 524
Antimon, crud........................99, 144,
145,373,382,385,431, 500,501,558, 579
Antimon, tart..........99,100, 154, 258,326,
381, 382, 383, 384, 385, 418, 431, 434, 498
Apis, Mel................-.....-......66,144,
159,195, 214, 313, 373, 385, 396, 426, 431
Apocyn....................-................„ 506
Aralia Rac.........................395, 506,590, 591
Aranea Diad...........-................—.264,	434
Arbutus, trailing................................. 486
Argent, met....................................382, 498
Argent, nit....... .....149, 211, 237,420,450, 451
Arnica...................................32,
68, 99,163, 213,214, 312,425,4,26, 432,
436, 498, 504, 527, 552, 553, 571, 601
.Arsenicum...—65,97,98, 99,100,144,145,
146, 147, 154, 156, 158, 159, 161, 162,
174, 175, 198, 264, 265, 313, 348, 355,
364, 373, 374, 375, 382, 383, 384, 385,
390, 407, 408, 409, 417, 418, 419, 420,
423, 424, 432, 490, 491,492, 494, 505, 601
Arsenic. Iodid..........................219,420, 425
Asa fet.............................381, 384, 600
Page.
Asterias....................................434
Astbmatos Cil....................................... 527
Atropia sulph..................................144, 520
Aurum...................................-406, 585, 586
Baptisia..............................152,419,432,579
Baptisin.....................................-.420
Baryta Carb......146,372,383,384, 394,432, 500
Bayard, Edward, M. D. Constantine
Hering..................................	10
Bayard, Edward, M. D. Cerebro-spinal
Meningitis in a Horse Cured by C.
M. potency..................................... 208
Bell—Croup. Walter M. James, M. D... 385
Bell..95, 97,99,110,114,144,145,154, 155,
158, 159, 161, 237, 265,312, 313, 322,
335, 343, 344, 346, 374, 381, 384, 385,
388, 390, 394, 406, 407, 409, 418, 419,
420, 422, 432, 443, 453, 454, 493, 498,
505, 520, 552, 579, 595, 598, 599, 601
Benzoic Acid..................................385, 432
Berberis-....................................432
Bernard,! Dr. On the Alternation of
Medicines. 408
Berridge, E. W., M. D. My Address at
Milwaukeee.................................... 46
Berridge, E. W., M. D. Clinical Cases,
67,117, 146, 376,446, 496
Berridge, E. W., M. D. A Case of Hy-
drophobia...................................334
Berridge, E. W., M. D. The Memorial
Volume______________................................. 365
Berridge, E. W., M. D. The Scientific
Use of the Nosodes........................ 516
Binghamton Homoeopathic Medical
Association, The. A. J. Clark,
M. D.............—-............................ 439
Boracic Acid........................................ 160
Borax.............144,145,374, 385,432,445, 446
Bovista......-....—......381,383,432,434, 497
Bristowe, John Syer, M. D. Address in
Medicine.—....—.......................... 510
British Medical Association and Ho-
moeopathy. Editorial........................509
Brom.........100,313, 382, 432
Bronchitis..............—...................597
Brown, D. Dyce, M. An M. D. On the
Treatment of Common Metritis,
especially that Form known as
Endo-Cervicitis, with Ulceration
of the Cervix...............................421
Bryonia........60,110, 113, 153, 154, 160,
213, 264, 160, 213, 264. 266, 268, 312,
313, 322, 324, 335, 369, 374, 382, 384,
409, 419, 432, 436, 452, 453, 492, 494,
495, 497, 498, 502, 527, 554, 579, 599, 601
Bufo.......................:................432
Burritt, Frances, M. D. A Case of Kid-
ney and Bladder Irritation Cured
by Syphilinum.............................. 120
Butler, C. W., M. D. Two Cases of In-
termittent Fever............................ 387
Cactus G289, 385,432,433, 434
Cainca......................................365
Calad.................................................., 433
Page.
Calameris Venenosus rubra..........444
Calatropis G,......................216
Calc. Carb....63,73, 153,158, 254, 255,
256, 285, 312, 338, 339, 342, 372, 374,
382, 383, 384, 410, 419, 423, 426, 433,
435 445, 454, 493, 497, 498, 506, 518, 600
Calc, fluor........................495
Calc, phos.....................„160,	579
Calendula: Its Place in Homoeopathic
Therapeutics. C. Carleton Smith... 31
Calendula............31, 32, 33, 34, 309, 422
Camphor......................161,162, 418
Cannab. Ind................ - ......
265, 383, 394, 446, 447, 496, 497, 518
Cann. sat„......................394,	447
Cantharis............200, 265, 381, 385,
418, 419, 450, 451,	505,	526,	554, 556,	557
Caps.......144,	145,	375,	382,	384, 419,	426
Carbo An....68, 372,	373,	382,	433, 447.	579
Carbo Veg......................144,
198, 212, 258, 265. 285, 340, 372, 375,.
381,	382, 383, 384, 419, 433, 497, 498, 504
Carbolic Acid....................35,	495
Carfrse, Geo. M., M. D. On the Treat-
ment of some of the Affections of
the Cervix Uteri........................423
Caries of the Jaw. Geo. G. Gale, M. D.. 67
Cariesin...........................529
Carlsbad Water.....................266
Carter, P. W., M. D. Indian Dysentery
and Cholera...................  418
Case of Hydrophobia, A. E. W. Ber-
ridge, M. D.....................334
Cases from Practice. D. C. McLaren,
M. D........................... 597
Castoreum..........................499
Catarrhal Diseases of Nasal and Res-
piratory Organs, by G. N. Brigham,
M. D. Review of.................164
Caust........145, 307, 313, 372, 373, 376,
382,	384, 394, 433, 451, 452, 498, 553, 600
Ceanothus.....-..........................216
Cedron.......„.....................264
Cepa...................................  433
Cerebro-spinal Meningitis in a Horse
cured by a C M Potency. E. Bay-
ard, M. D...............-.......208
Cervix Uteri: On the Treatment of
Some of the Affections of the.
Geo. M. Car free, M. D..........423
Cham....„............100,213,312,313,
382,	384, 433, 434, 436, 445, 447, 498, 553
Change of Heart....................458
ChargADr. A. Hemorrhoids and Their
Treatment................141,188, 372
Chelidon.......................433,	435
Cheney, B. H., M. D. Is Hahnemann
Obsolete?....................... 90
China. ...................... --GO,
97, 99, 154, 234, 265, 313. 372, 375,
383,	384, 385, 390, 394, 418, 433, 449, 498
Chloroform.......................  426
Chlorum...........-.........'......497
Cicuta...........................  420
Cina.......—..............99,100,159,
261, 312, 384, 386, 387, 392, 433, 498, 600
Cinchon.....................407, 419, 530
Cinnabar...........................107
Clstus.........................    385
Clapp, H. C., M. D. “ Is Consumption
Contagious?”	Review of........ 78
Clark, A. J., M. D. The Binghamton
Homoeopathic Medical Associa-
tion..........................  439
Clark, George H., M. D. Intermittent
Fever with Cases................173
Clark, George H., M. D. The False and
the True: A Warning.............332
Clem, erecta....................382,	451
Page.
Clinical Bureau......................
326, 338, 376,445, 490, 551, 591
Clinical Cases. E. W. Berridge, M. D....
67, 146,376,446, 496
Clinical Cases. W. A. Hawley, M. D... 195
Clinical Cases. E. B. Nash, M. D..... 152
Clinical Cases, with Comments. C.
Pearson, M. D................... 197
Clinical Cases: Chronic Disease Cured,
Thomas Skinner, M. D............. 254
Clinical Cases Cured with Metals. Wm.
P. Wesselhoeft, M. D.............326
Clinical Cases. Edw. Rushmore, M. D. 338
Clinical'Cases. John F. Miller, M. D.... 348
Clinical Reflections. Ad. Lippe, M. D.
110, 587
Cocc„............382, 383, 384, 433,498, 579
Cod.......  ......................... 385
Coff................................. 385
Colch................144, 381, 385, 419, 433
Colic............................... 380
Collins......................144,145, 192
Coloeynth.................66,144, 215,
322, 372, 381, 382, 418, 433, 437, 505, 570
Comocladia...„.......................420
Confinement, Ailments after.......... 496
Conium................146, 382,383, 393, 420
Conservative Surgery. H. I. Ostrom,
M. D........................... 305
Constipation.........................152
Constipation, Chronic. E. B. Nash,
M. D............................. 69
Consumption Contagious,Is. Review
By Herbert C. Clapp, M. D. A Re-
view of.......................... 78
Corn................................  385
Coms.................................500
Coto Bark............................453
Cough........................340, 376, 498
Creosote.....................144, 261, 263
Cretin, Dr. The Question of Doses;
Hahnemann and Homoepathy........ 415
Crocus................382, 394,433,498, 600
Crotalus..................•......417,	426
Croton......„................385, 418, 522
Croup.....................  -........ 385
Cub.................................. 385
Cup. acct............................ 418
Cupr. Metal.........................  312
326, 327. 328, 343, 406, 433, 484, 505
Curious Case, A:—Cantharis. Wm. A.
Hawley, M. D........—.............. 554
Cyclamen...........-.......-.....219,	434
Dake, J. P., M. D. Drug Attenuation.
Its Influence upon Drug Matter and
Drug Power........................... 411
Deafness............................. 149
Diarrhoea........................445.	449
Diarrhoea, Homoeopathic Therapeutics
of: by J. B. Bell, M. D. A Review of 396
Differential Diagnosis of Merc. Iod.
Flav. and Merc. Iod. Ruber, in
Diphtheria. C. Carleton Smith,
M. ..............................105
Digitalis.....................  155,
163, 313, 382, 384, 418, 434, 498, 559. 579
Dios.............-..........—........ 385
Diphtheria.......................350.	598
Diphtheria, by R. R. Gregg, M. D. A
Review of........................ 37
Diphtheria, A Treatise on: by A.
McNeil, M. D. A Review of........398
Diphtheria: Differential Diagnosis be-
tween Merc. Iod. flav. and Merc.
Iod. Rub. in. C. Carleton Smith,
M. D .........J.................. 105
Diphtheria and Bacteria. P. P. Wells,
M.  .........................—•« 239
Page.
Diphtheria and Bacteria. Rollin R.
Gregg, M. D.................................... 474
Diphtheria, Bacteria and Dr. Gregg: P.
P. Wells, M. D................................ 546
Diphtheria, Bacteria and Dr. Wells: R.
R. Gregg, M. D............................... 571
Discovery, A New, in the Service of
Homoeopathy. Trans, by Prof.
Manuel Graeter............................538,	580
Disease of Infants. C. Lippe, M. D.............312
Diseases peculiar to Infants and Chil-
dren, A Treatise on: W. A. Ed-
monds, M. D. A Review of.......................396
Doses: The Question of: Hahnemann
and Homoeopathy. Dr. Cretin-------------------- 415
Dropsy from Malaria. W. H. Jenny.... 64
Droseria.......................265. 352,382. 383, 498
Drug Attenuation: Its Influence upon
Drug Matter and Drug Power. J. P.
Dake, M. D..........___________I............... 411
Drug Proving. Ad. Lippe, M. D.......... 287
Duboisin'.......................................262
Dulc..........................................265,385, 434
Dysentery. Carbo v..............................198
Editorials............................41,167, 509, 561
International Hahnemannian
Association........................... 223
Law of the Similars..................... 81
Liberty of Medical Opinion and
Action................................123
Reply to “Instruction not En-
dorsement”............................... 254
Salutatory............................... 1
Study of the Materia Medica....... 96
Elaps Corallinus...........................417,	426
Elat..................................................... 385
Endorsement...................................  181
Epigcea. By C. F. Millspaugh, M. D.... 486
Erigeron.....................................   144
Errata................................166,400, 604
Erythrox.............................................. 447
Eucalyptus  ....................................162
Eupator, purp.....<..............159,352, 388, 389
Euphor.......................................   383
Euphrasia.............................333, 382, 434
Euthanasia. Geo. G. Gale, M. D.......... 66
False Accusation, A.............................550
False and the True, The: A Warning.
Geo. H. Clark, M. D........................ 332
Fatal Errors. A. Lippe.............47, 87,139,
171,230, 363, 428.467, 528
Fellger. Ad., M. D. Homoeopathy......	22
Magna est Veritas et Praevalebit.. 316
Ferrum............144, 382, 383,384, 394,424, 498
Ferrum Phos................................214,	493
Field: The: Letter to Editor of. D.
Wilson, M. D  ............................ 203
Fifth Annual Session of the Missouri
Inst, of Homceop................................362
Fluxion Dilutions. Wm. Jefferson
Guernsey. M. D.........................236, 471
E. C. Price, M. D.......................... 366
Following Pickwick’s Advice............... 489
Foote, Geo. F.,M.D. Similia Similibus
Curantur....................................... 314
Formica  ..................................... 426
Fracture of Fourth Metacarpal Bone... 308
Fracture of Coccyx.............................. 307
Gale, Geo. G., M. D. Euthanasia......... 66
Caries of the Jaw....................... 67
Gamboge..................................  384,	385
Gastralgia nux v. Carbo v.......................212
Gelsem...............................154,158,
175. 419, 424, 426, 434, 503, 504, 579, 603
Gem, A................................................. 160
Page.
Gemiasma Verdans.............................. 466
Generalization and Individualization.
R. Hughes................................	405
Genito-urinary Therapeutics................ 599
Gleet..................................................... 450
Glon........................................  313,	579
Greater, Prof. M.: A New Discovery in
the Service of Homoeopathy—
Translation..............................538, 580
Graphi...........144,145,159. 209, 285, 349, 381
383, 385, 423, 434, 436, 450, 455, 495
Grat..........................................,........... 385
Gregg, R. R., M. D. Disease of the Hoof
of the Horse........................................208
Superiority of High over Low
Potencies................................. 341
Diphtheria and Bacteria.....................474
Diphtheria, Bacteria and Dr.
Wells........................................ 571
Guajac................................................. 394
Guernsey, Wm. Jefferson, M, D., Flux-
ion Dilutions............................236, 471
Guide to Clinical Examination: By
Richard Hagan, M. D. Review of. 270
Guiding Symptoms of Our Materia
Medica, The: by C. Hering. Re.-
view of........................................... 559
Hamamelis..............................144, 420, 424
Hawley, Wm. A., M. D., Clinical Cases. 195
Inflammatory Rheumatism......... 386
A Curious Case............................ 554
Hayle, Thos., M. D., Thoughts on the
Scientific Application of the Prin-
ciples of Homoeopathy in Practice. 403
Headache............................................. 497
Hellebore........................ 154, 312, 313
343, 344. 345, 346, 347, 382, 394, 490
Helonias..................................424, 570, 571
Hemorrhage, Post Partem..............-...... 446
Hemorrhoids..................................146. 499
Hemorrhoidsand Prolapsus................ 148
Hemorrhoids and their Treatment. Dr.
A. Charge.........................141, 188, 372
Hepar. S. C............144, 145, 154, 160, 216,
261, 265, 313, 382, 384. 409, 431. 434,
453, 493, 498 506, 558, 598, 599, 600, 601
Hering Constantine. By Edward Bay-
ard, M. D....................................... 10
Hering Memorial : By the Memorial
Committee................................... 56
The: A Circular............................108
High Potencies in Parturition. Julius
Schmitt, M. D................................. 551
Historian, The: The Penn. Medical
University...................................... 370
Homoeopathic Achievements............... 165
Homoeopathic Physician’s Visiting List
and Pocket Repertory. By Rob't
Faulkner, M. D. Review of......................... 603
| Homoeopathic Therapeutics as applied
to Obstetrics. By Sheldon Leavitt,
M. D. Review of.......................... 559
i Homoeopathic Treatment of Diph-
theria, The. By DeForest Hunt,
M. D. Review of............................. 603
Homoeopathy. A Fellger................... 22
Homoeopathy: What Is? C. H. Law-
ton, M. D.....................................562
Homoeopathy in the Treatment of Dis-
eases Prevalent in India. Mahen-
dra Lal Sircar. M. D....................... 418
I Honor to Whom Honor is Due. E. J. L. 35
I Holcombe, W. H., M. D. On the Differ-
. ential Diagnosis and Treatment of
Yellow Fever................................. 416
Horse: Cerebro-Spinal Meningitis Im
E. Bayard. M.J)................................208
Page.
Horse: Hoof of the: Disease of the
R. R. Gregg, M. D............. 205
Hospital for Incurables........... 165
How to See with the Microscope. By
J. Edwards Smith, M. D. Review of 272
Hughes, R. L. R. C. P. Generalization
and Individualization..............405
Hydrastis......145.	420, 422, 425, 426, 579
Hydrocy. Ac.................   312,	418
Hydrophobia....................... 334
Hydrophob...............334, 336, 395, 527
Hyos....................154, 155. 158,
322. 343, 344, 383, 384, 385, 394, 418, 498
Hypericum Perfol...........263,	392, 425
Ignatia.................100, 144, 145,
381, 382.. 423, 426, 434, 455, 498, 505, 579
Indian Dysentery and Cholera. P. W.
Carter, M. D...................418
Indigestion....................... 340
Inflammatory Rheumatism—Cina. W.
A. Hawley, M. D............... 386
Instruction not Endorsement, T. F.
Pomeroy, M. D................. 252
Reply to: Editor.............. 254
Intermittent Fever, with cases, Geo. H.
Clark. M. D................ 173
Two Cases of: C. W. Butler, M. D. 387
E. W. Rushmore, M. D..........490
“International”: The Observer: In-
troductory........................ 184
International Hahnemannian Associa-
tion.......................79,	164
Editorial.....................223
T. F. Pomeroy, M. D.......... 437
Homoeopathic Convention, 1881.. 401
Homoeopathic Convention of’81.
The. P. P. Wells. M. D..... 459
Iod.............313,	382, 383, 385, 432, 434
Ipecac    ........ ...............144,
”154,''382/384‘ '39<)/419/498,' 579’, 601
Iris Vers......................263,	385
Is Hahnemann Obsolete? B. H. Che-
ney, M.	D..................... 90
Isopathy; A Fatal Error. Ad. Lippe,
M. D............................ 528
Items..............................165
Jaeger: Has	Not Heard of...*.......222
'Jalap......................    216,	261
James, Walter M., M. D. Review of
“Medical Heresies.”........... 71
Quinsy........................114
Bell.—Croup.................. 385
Jatropha.......................385,	434
Jenny, W. H., M. D. Dropsy from Ma-
laria.............................. 64
Kali Bichrom.................. 68,
313, 372, 373, 385, 394, 395, 418, 432, 591
Kali-Brom........................  434
Kali-Carb...100, 117. 118, 119, 120, 145,
146, 198, 238, 255, 256, 265. 330, 339,
381, 382, 384, 435, 449, 496, 498, 558, 570
in Rheumatism. E. W. Berridge,
M. D....................... 117
Kali Iod...........................505
Kalmia............................ 383
Key-notes, Some. C. Carleton Smith,
M. D.......................... 431
Kidney and Bladder Irritation cured
by Syphilinum. By Frances Bur-
ritr, M. D.........................120
Lachesis..................... 68,	69,
116, 145, 158, 159, 162, 163, 196, 215,
234, 237, 262, 289, 290, 292, 313, 322,
335, 348, 349, 372, 373, 382, 383, 389,
394, 417, 419, 426, 435, 456, 503, 505, 591
Page.
Lachnantes.................... 212,	291
Lac Caninum................ 69, 350., 351
Lac Vaecinum Defloratum............ 69
Lactic Acid............ 263, 264, 324, 393
Lact.............................. 385
Laur.............  381,	382, 384, 497, 498
Lawton, C. H., M. D. What is Homoeo-
pathy?...........................  562
Law of the Similars; The: Editorial... 81
The: Is This All ? C. Pearson. 29
Lectures, Clinical and Didactic, on the
Diseases of Women. By R. Lud-
lam, M. D., Review of....;.........456
Ledum......................... 145,	384
Lee, E. J., M. D. Pathological Pre-
scribing: A Science Falsely So-
called.............................319
L., E. J. Honor to Whom Honor is
Due.......................   35
Notes from our Scrap-Book.... 579
Legion of Honor, The. A. Lippe..... 44
Legitimate Homoeopathy. C. Pearson,
M. D........................... 351
Lept.............................. 385
Letter, An Open. T. F. Pomeroy, M.D. 137
Liberty of Medical Opinion and Action.
Editorial.......................123
Lilium Tig..........198,	423, 424,432, 579
Lippe, Ad., M. D. Clinical Reflections.
110, 587
Drug Proving..................	287
Fatal Errors..................
47, 87,139, 171, 230, 363,428, 467
Isopathy: A Fatal Error.......528
In Memoriam................... 3
Legon of Honor................. 44
Medical Heresies: A Fatal Error. 47
The Penn Medical University:
A Fatal Error...............428
Review of Gregg on Diphtheria.. 37
Review of Transactions of Amer.
Inst. Hom., 1880........... 38
Third Paragraph of Hahne-
mann’s Organon................131
Lippe, C., M. D. Similia vs. Idem.. 94
Diseases of Infants........... 312
Li th. ac.......................... 385
Lyc..........116,144, 145,159,201, 213,
215, 234, 257, 312, 334, 338, 339, 347,
375, 382, 383, 384, 394, 419, 435, 450,
. 493, 498, 503, 518, 553, 601
Lyssin..........................   524
Macfarlan, John, M. D. Puerperal
Mania with History of a Case........ 591
Magna Est Veritas et Prsevalebit. Ad.
Fellger, M. D.................. 319
Magnesia carb......................381
382, 383, 384, 385,432, 497, 498
Magnes. mur......99, 881, 384, 435, 498, 579
phos....................  214,	493
sulph.........................497
Magnet......................379, 382. 384
Majumba. Pratap Chandon, L. M. S.
Malarious Fever in India.......419
Malarious Fever in India. Pratap
Chandon, Majumba, L. M. S....... 419
Mang...........................381,	383
Mangan, acet.......................435
Manganum Carb..................... 380
Martmy, Dr. On the Alternation, of
Medicines.....................  408
MarumV.............................159
McLaren, D. C., M. D. Cases from
Practice......v.................597
Medical Heresies...................165
Medical Heresies, by Gonzalvo C.
Smythe, M. D. Review of....... 71
Page,
Medical Heresies. A Fatal Error. A.
Lippe,;M. D................. 	47
Medicine: Address in. John Syer
Btistowe, M. D........................  510
Medorrhinum~....162, 365. 503, 525, 526, 532
Meeting of the International Hahne-
mannian Association. Report by
Edward Rushmore, M. D.......................273
Meg......................................   382
Melancholia, Post Partem...................„	147
Melitagrinum..............................  527
Memorial Volume, The. E. W. Ber-
ridge, M. D______........ _................ 365
Memoriam, In.	By A. Lippe................. 3
Menyan.......|.............................. 381
Meph......................................  579
Mercurius...97.99,107,115,121, 144, 145,
214, 313, 322, 381, 383, 409, 419, 423,
424. 450, 451, 452, 454, 497, 523, 601
Merc. Corr. Sub..............107,	198, 419, 435
Merc. Iod. Flav. and
Merc. Iod. Rub. Differential Diagnosis
between, in Diphtheria. C. Carle-
ton Smith, M. D.,.......................... 105
Merc. Iodat. Rub...............107, 116,156, 579
Merc. Precip. Rub.........................  268
Merc. Sol.........................................154,
265, 387, 390, 394, 419, 435, 495, 506
Merc. Sulphate.................................... 107
Merc. Viv. and Podophyl. Differential
Diagnosis between. C. Carleton
Smith, M. D................................... 57
Metritis: On The Common Treatment
of, especially that Form known as
Endo-cervicitis with Ulceration of
the Cervix. D. Dyce Brown, M. A.,
M. D............................................. 421
Mez.............................383,384, 385,450, 602
Millefol................................144,	313
Miller, John F., M. D. Clinical Cases. 348
Millspaugh. C. F., M. D. Potency
Physically Considered.................. 440
Millspaugh, C. F„ M. D. Epigsea............486
Monotropa U.................................263
Moschus.......................384,496, 497, 498
Muriatic Acid .....................144,	145, 313
323, 348, 393, 419 ,435, 449, 450, 579
NajaTripudians........................  417,	426
Naphthaline..........	263
Nash, E. B., M. D., Chronic Constipation 69
Clinical Cases.......................152
Podophyl, jand Borax in Diar-
rhoea........................................ 445
Nat. carb..................................381,383, 385
Nat. Hypochlor..............................447
Nat. mur..................................145,146,
158, 159, 174, 268, 285, 329, 330, 372,
373, 376, 378, 379, 381, 382, 383, 394,
450, 491, 497. 498, 557, 579, 584, 585, 601
Necrosis of Os Calcis...................... 309
Nervous' System, Diseases of: C. P.
Hart, M D. A Review of............... 397
Neuralgia.............................................. 376
Niccol.............................................385, 579
Niccol: Sulph.............................. 211
Nichols, C. F., M. D., Quantum Sufficit... 52
Acrochordon Chocoe: Charac-
teristics.................................... 444
Rheumatism...........................491
Nitric, acid...................121,144,145,
214, 324, 325, 381, 383, 384, 393, 451, 591
Nitrogen-ox.................................447
Notes on Current Literature.................507
Notes From Our Scrap-Book. E. J. L... 578
Notices, Comments, &c......................... 222
Nux Moschata...............50. 258,381,385, 432
Page.
Nux Vom..........................50.95, 97,144
145, 153, 154, 158, 159, 162, 208 212,
213, 219, 261, 263, 322, 340, 342’ 343,
344, 346, 347, 352, 374, 375. 381, 382.
384, 385, 390, 393, 407, 409, 410, 419,
423, 424, 432, 435, 450, 498, 500, 504,
506, 522, 552, 553, 556, 557, 597, 601, 602
Observer’s Introductory for 1881, The
“International”............................. 135
Observer, The: Introductory “ Inter-
national”......................................... 184
(Enanthe Crocato.................................. 559
Oleander.................................384, 394, 436
Ophthalmia.........................................376
Opium...98.159, 235,268,384,385,436,498, 505
Organon The: Section 153, P. P. Wells,
M. D............................................... 15
Ostrom, H. I, M. D., Conservative
Surgery........................................... 305
Oxalic add..................................  393,	436
Paris Quad.........................................434
Parturition...........1............ .................. 551
Passifloria incarn................................. 453
Pathological Prescribing A Science
Falsely So-Called. E. J. Lee. M. D”. 319
Pearson, C., M. D., Clinical Cases with
Comments.................................... 197
The Law, Is this All............................? 29
Legitimate Homoeopathy............. 351
The What Is It............................. 60
Penn Medical University, The: The
Historian................................... 370
A Fatal Error, Ad. Lippe, M.
D....................... .............  428
The:466
Periostitis of Femur.............................. 308
Periscope...............................154, 211,
259, 390.452, 501,' 557, 600
Perverted: The History of Homoe-
opathy, editorial............................ 167
Petiv..............................................447
Petrol..l45,156,161, 382,436,451,496,497, 579
Philosolphy of Homoeopathy. P. P.
Wells, M. D,.........„.	277
Phosphorus............50, 67, 99.144,145,146,
148, 149, 150, 159, 213, 215, 239, 265,
268, 269, 313, 324, 339, 340, 351, 369,
373, 381, 382, 383, 384, 385, 395, 417,
418, 420, 424, 426, 436. 455, 498, 579, 588
Phos. ac....l44, 372, 375,381,384, 394,419, 436
Physicians’ Quarterly Record and State-
ment; by Wm. Jefferson Guernsey,
M. D. Review of........................... 603
Phytolacca.........................106,313,424, 436
Pickwick’s Advice, Following.............. 489
Picric acid......................................376, 426
Platina.......144,329, 330,381,423,432,497, 517
Plea for a Standard Limit of Attenu-
ated Doses, A, C. Wesselhceft, M. D.. 414
Pleurodynia......................................... 214
Pleuro-Pneumonia..........................112, 113
Plumb. Acet....................................... 436
Plumb, met...,..............312,328,381,434, 579
Pneumonia. Lyc. D. Wilson, M. D.... 201
Podophyllum...................57,144,145,198,
213,219, 385, 445
Podophyllum and Borax in Diarrhoea.
E. B. Nash, M. D............................ 445
Pomeroy. T. F., M. D., Instruction not
Endorsement............................ 252
The International Hahneman-
nian Assodation...................... 437
An Open Letter............................. 137
Postpartem Complaints........................ 496
Potency Physically Considered. C. F.
Millspaugh, M. D............................ 440
Page, i
Practice: Cases from: D. C. McLaren,
M. D............................ 597
President Garfield, Treatment of: T.
Dwight Stow. M. D............... 533
Prevention of Congenital Malforma-
tions by Medicinal and Nutritional
Treatment. A Review of............. 271
Price, E. C., M. D. Fluxion Dilutions.. 366
Prolapsus and Hemorrhoids.......... 148
Prolapse of Hemorrhoidal Tumor and
Rectum......................... 348
Proving of Ammo. Carb. Leila A.
Rendell, M. D...................292
Psoriastnum.......................... 528
Psoric Irritation of Eye and Spine.. 338
Psorinum..............58,158, 217, 328,
372, 385. 391, 392, 393, 436, 523, 527, 530 |
Puerperal Mania—with History of a
Case. John Macfarlan, M. D.......591
Pulsatilla..98,99,100.144,145,153.159,160,
210, 214, 262, 265, 307, 311, 313, 322,
345, 368, 373, 375, 381, 382, 383, 384,
385, 394, 418, 422, 424. 473, 492, 495,
498, 500, 504, 505, 506, 507, 551, 552, 600
Qaantum Sufficit. C. F. Nichols, M. D. 52
Question Answered, The: C. Carleton
Smith, M. D..................... 232
Quinine sulphate........264,	419, 426, 433
Quinsy by W. M. James, M. D........ 114
Ranunculus Bulb..................436,	590
Seel........381,420,500,	501
Raph...,..............................385
Ratanhia........................145,	214
Remedies, Unproved, and Dr. Berridge.
P. P. Wells, M. D.............. 566
Rendell, Leila A., M. D. Proving of
Ammo. Carb......................292
Reviews, Book Notices, &c......37, 71.
164, 270, 396, 456, 507, 559, 603
Rhad............................   385
Rheumatism..................... 117,	491
C. F. Nichols, M. D...........491
Rheum...........................312,	436
Rhod............146,	381, 385, 419 434, 437
Rhus Aromat........................557
Rhus Tox....;.........58,	98, 99, 111,
144, 155, 158, 179, 262, 264, 265, 266,
307, 312, 313, 322, 373, 382, 383, 384,
387, 394, 419, 426, 436, 450, 451, 453,
490, 494, 495, 498, 502, 503, 506, 600, 602
Robinia, p. a......................393
Rumex C........................ 215,	385
Rushmore, Edward, M. D. Clinical
Cases.........................338
Intermittent Fever............490
Report of Meeting I. H.	A.....273
Ruta G......................... 384,	498
Sabadilla.......,.............. 381,	437
Sabina........................._	312, 424
Saccharum Off.................. 522,	525
Sad Case, A.....................   122
Salicylic Acid.......................426
Salicylicate of Soda................426
Salutatory.........................  1
Sambucus................327,	383, 384, 437
Sanguin..........................99,	579
Sarsaparilla................381,	437, 450
Schmitt, Julius, M. D. High Potencies
in Parturition................  551
Scientific Use of the Nosodes, The: E.
W. Berridge. M. D............... 516
Scill.......................... 382,	385
Scirrhinum........................ 52 7
Page.
Secal Corn.............364,	385. 418, 496
Sed. Teleph.....................   144
Selenium...........................215
Senega...........................  384
Sepia.......97,	145, 153, 256, 285, 312,
313, 372, 375, 381, 383, 384, 385, 396,
422, 424, 425, 435, 437, 497, 498, 557, 579
Sexual weakness................... 146
Silicea................69. 70, 71. 99,
144, 145, 149, 154, 158, 160, 215, 216,
268, 285, 369, 372, 375, 381, 382, 384,
391, 419, 433, 434, 435, 437, 495, 496, 600
Similia Similibus Curantur, Geo. F.
Foote, M. D.................... 314
Similia versus Idem, C. Lippe, M. D.... 94
Similia, A New, A. W. Woodward. M. D. 407
Sircar, Mahendra Lal, M. D. Homoeo-
pathy in the Treatment of Diseases
Prevalent in India......;..........418
Skinner, Thos. M. D. Cases of Chronic
Disease Cured...................254
Smith, C. C., M. D. Aconite in its Re-
lation to the Female Sexual Sys-
tem .............................. 569
Calendula; Its place in Homoeo-
pathic Therapeutics.......... 31
Differential Diagnosis between
Merc. Iod. Flav. and Merc. Iod.
Rub. in Diphtheria..........105
Differential Diagnosis of Merc.
V. and Podophyl............. 57
Some Key-notes................431
The Question Answered.........232
. Solan. Nig........................447
Sore Throat, Lach..................196
Spectacles and How to Choose Them.
By C. A. Vilas, M. D. Review of.... 270
Spigelia...............313,	322, 384, 498
Spongia........313, 381, 382, 383, 406, 409
Stannum........381, 382, 437, 579, 597, 598
Staphysagria................  381,
382, 383, 384, 394, 498, 579
Stictapul,........................ 155
Stow, T. Dwight, M. D. Treatment of
President Garfield............. 533
Stram...........312, 313, 394, 418, 496, 497
Stront............................ 381
Strychnia..............157,	214, 434, 522
Study of the Materia Medica. Editorial 96
Sulphur.......67, 99, 111, 115, 116, 145,
147, 148, 153, 159, 192, 198, 213, 216,
219, 238, 265, 266, 313, 328, 329, 334,
345, 375, 381, 383, 384, 385, 391, 392,
394, 409, 410, 422, 434, 445, 452, 493,
494, 495, 497, 498, 499, 500, 502, 503,
504, 518, 522, 523, 526, 532, 558, '571, 591
Sulphuric Acid..............;.... 154,
310, 373, 382, 383, 384, 385, 393
Surgical Principles and Minor Surgery.
By J. G. Gilchrist, M. D. Review of 271
Superiority of the High over the Low
Potencies in the Treatment of the
Worst Forms of Disease. R. R.
Gregg, M. D...................  341
Symphytum O.. „................... 308
Syphilinum...........  120,	121, 122,
334, 360, 365. 503. 519, 520, 522, 526, 530
A Case of Kidney and Bladder
Irritation Cured by. Frances
Burritt, M. D.................120
Tartar Emet................ 313, 417, 436
Terebinth..................215, 418, 601
Thea...............................497
Theridion......................... 291
Third Paragraph of Hahnemann’s
“Organon” with Comments. A.
Lippe, M.D............,........ 131
Page.
Thoughts on the Scientific Application
of the Principles of Homoeopathy
in Practice. Thomas Hayle, M. D. 403
Thuja................................... 158,	159,
216, 257, 264, 265, 376, 377, 381, 385,
450, 493, 496' 526, 583, 584, 5S5, 586, 600
Tonsillitis........................................ 195
Transactions of the American Institute
of Homoeopathy 1880. Review of... 37
Transactions of The World’s Homoeo-
pathic Convention of 1876. A Re-
view of..............-............................. 164
Treatise on The Decline of Manhood;
Its Causes and the Best Means of
Preventing their Effects. By A. E.
Small, M. I>. A Review of.............. 458
Treatment of President Garfield T.
Dwight Stow, M. D........................ 533
> Trilli pend......'.................................. 144
Trumpet Weed..................................... 390
Tuberculinum................................   527,	529
Typhoid Fever........................... 110,	111, 152
Unproved Remedies, P. P. Wells, M. D. 357
Unproved Remedies and Dr. Berridge
P. P. Wells, M. D............................ 566
UsneaBarb.......................................... 452
'Uterus. Displacement of. Thos. Skin-
ner, M. D....................................... 254
Uterus. Prolapsus of................................256
Viola Od...................................... 312
Vipera Torva...................................... 417
Vaecinum Defloration......................... 69
Variolinum.................... ..............	522, 527
Verat. Alb...........................................
153. 161, 162, 163, 219, 369, 373, 375,
382 383, 384. 385, 406, 407, 418, 498
Verat. Vir......................................... 602
Verb..................................................... 381
Verrugosa Acrochordon Chocoe .......... 444
Page.
Vespa................................................... 426
Veterinary Cases.................................. 201
Viburnum Opulus. A Fragmentary
Proving. H. C. Allen, M. D........... 101
Wanted....................................... 458,	466
Wells, L. B., M. D. Abnormalities of
Labor....................................... 310
Address: “The Philosophy of
Homoeopathy.”............................277
Diphtheria and Bacteria............ 239
Diphtheria, Bacteria and Dr.
Gregg546
The International Homoeopathic
Convention of 1881...................... 459
Organon Sec. 153........................ 15
Unproved- Remedies..........................357
Unproved Remedies and Dr.
Berridge................................... 566
Wesselhoeft, C.’ M. D. A Plea for a
Standard Limit of Attenuated
Doses............................................. 414
i Wesselhoeft, Wm. P., M. D. Cases cured
with Metals.................................. 326
I What Is It. The. C. Pearson............. 60
i Wilson, D„ M. D. Letter to Editor of
Field....................................203
Lye.in Pneumonia....................:. 201
Winter Cough................................ 381, 382
Woodward, A. W., M. D. A New Sim-
ilia.....................-.............................. 407
Xanthoxylnm...................................... 424
Yellow Fever: On the Differential
Diagnsis and Treatment of: W. H.
Holcombe, M. D.....................................416
Zinc. Met...................................50, 312,
381, 382, 384, 394, 422, 455, 493, 498
Sulph ......................................... 214
CONTRIBUTORS:
Drs. H. C. Allen, T. F. Allen,- Edward Bayard, J. B. Bell, E. W. Berridge,
Geo. H. Clark, A. C. Cowperthwaite,, Ad. Fellger, Wm. J. Guernsey,
W. A. Hawley, John Hall, W. M. James, W. H. Jenny, Ad. Lippe,
C. Lippe, Malcolm MacFarlan, Thos. Moore, C. F. Nichols,
C. Pearson, T. F. Pomeroy, Leila A. Rendell, C. Carle-
ton Smith, P. P. Wells, Wm. P. Wesselhoeft,
David Wilson, (London), T. P. Wilson,
and many others.
NOTICE.
All articles for publication, books for review, business communications, etc.,
must be sent to Editor, 2100 Chestnut Street, Philadelphia.
N. B.—Authors of accepted papers are furnished with copies oi
the number in which their, articles appear; application for' such
copies to be made when sending papers. Any irregularity in the
receipt of this Journal should be promptly reported to Editor.
A prize of fifty dollars will be awarded to author of the best
essay printed in this Journal for the year 1881. To be adjudged
by three physicians of note
“Subscribers who have not paid their Subscrip-
tions will please give the matter their
prompt attention/..
				

